# MicroRNAs in Alzheimer’s Disease

**DOI:** 10.3389/fgene.2019.00153

**Published:** 2019-03-01

**Authors:** Mengli Wang, Lixia Qin, Beisha Tang

**Affiliations:** ^1^Department of Neurology, Xiangya Hospital, Central South University, Changsha, China; ^2^Laboratory of Medical Genetics, Central South University, Changsha, China; ^3^National Clinical Research Center for Geriatric Disorders, Changsha, China; ^4^Key Laboratory of Hunan Province in Neurodegenerative Disorders, Central South University, Changsha, China; ^5^Parkinson’s Disease Center of Beijing Institute for Brain Disorders, Beijing, China

**Keywords:** Alzheimer’s disease, microRNAs, pathological process, amyloid plaques, neurofibrillary tangles, Aβ, Tau

## Abstract

Alzheimer’s disease (AD) is a progressive and devastating neurodegenerative disorder. It is the leading cause of dementia in the world’s rapidly growing aging population. The characteristics of AD are memory loss and cognitive impairment, meaning patients cannot carry out their daily activities independently. The increase of AD cases poses heavy burdens on families, society and the economy. Despite frequent efforts being made to research the etiology of AD, the causes of AD remain unknown, and no curative treatments are available yet. The pathological hallmarks of AD are amyloid plaques and neurofibrillary tangles in the brain. MicroRNAs are endogenous ∼22 nucleotides non-coding RNAs that could regulate gene expression at a post-transcriptional level by transcript degradation or translation repression. MicroRNAs are involved in many biological processes and diseases, particularly multifactorial diseases, providing an excellent tool with which to research the mechanisms of these diseases. AD is a multifactorial disorder, and accumulating evidence shows that microRNAs play a critical role in the pathogenesis of AD. In this review, we will highlight the effect of microRNAs in different pathological processes throughout AD progression.

## Introduction

**FIGURE 1:**
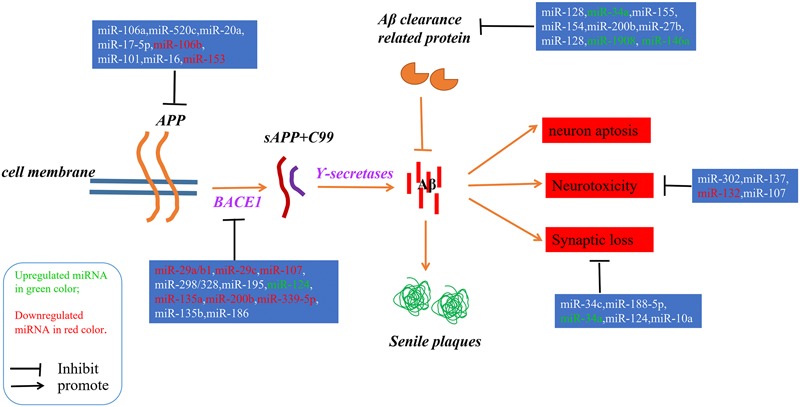
A schematic of the Aβ hypothesis of AD pathogenesis and the microRNAs involved in each step. The Aβs were produced as a result of processing the amyloid precursor protein (Richter et al., 2012) through a sequential enzyme digested by BACE1 and γ-secretase; an imbalance between the production and clearance of Aβ is the key trigger of AD.

**FIGURE 2:**
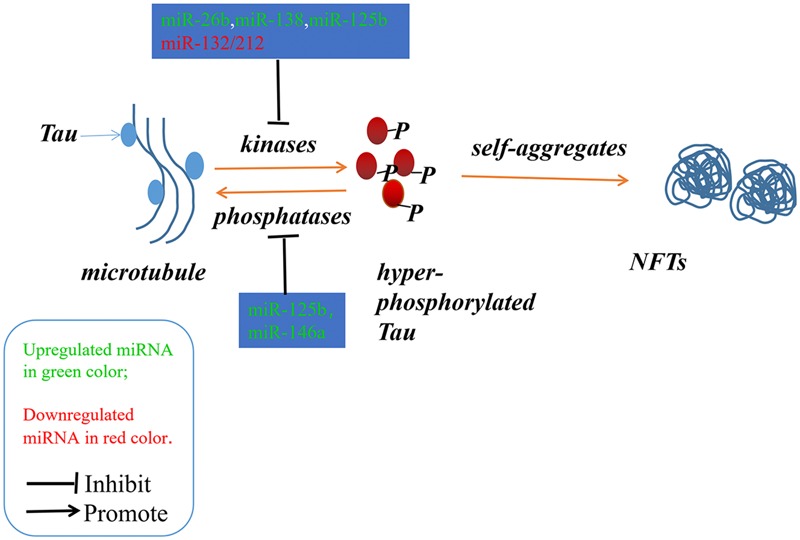
The imbalance between the hyper-phosphorylated and de-phosphorylated processes of Tau could lead to the formation of NFTs. The microRNAs involved in the phosphorylated and de-phosphorylated processes play a role in AD pathogenesis.

Alzheimer’s disease (AD) is an irreversible progressive neurodegenerative disorder, and is the main cause of dementia in the global elderly population. In 2010, it was estimated that there were 35.6 million people living with dementia across the globe; these numbers are expected to double every 20 years until 2050 (Prince et al., 2013). The characteristics of AD are cognitive impairment and memory loss, causing most AD patients to lose the ability to perform daily activities independently. The cost of caring for these patients is rising, with increasing numbers of AD incidents around the world, posing heavy burdens for individuals, families and society. Although mounting efforts have been made to research the etiology of AD, the causes remain unknown, and effective treatments are not yet available (Idda et al., 2018). The pathological hallmarks of AD are senile plaques consisting of accumulated β-amyloid peptides (Aβ) and neurofibrillary tangles (NFTs) primarily containing highly phosphorylated Tau (Ballard et al., 2011). The two most commonly accepted hypotheses, the Aβ hypothesis and the Tau hypothesis, are also based on these two pathological characteristics. The amyloid cascade hypothesis (see Figure 1) suggests that the imbalance between the production and clearance of Aβ is the key trigger of a cascade of events that leads to AD (Blennow et al., 2006; Ballard et al., 2011). The Aβs are produced from processing the amyloid precursor protein (Richter et al., 2012) through sequential enzymes digested by β-secretase (beta-site amyloid precursor protein cleaving enzyme1, BACE1) and γ-secretase (consisting of presenilin1 and presenilin2) (Querfurth and LaFerla, 2010). In physiology, Aβ-degrading proteases mediate proteolytic degradation and receptor-modulated endocytosis function to clear the Aβ (Hauser and Ryan, 2013). The proteolytic system includes neprilysin and an insulin-degrading enzyme (Qiu et al., 1998; Iwata et al., 2001; Kanemitsu et al., 2003); the low density lipoprotein receptor family is involved in the receptor system. The most toxic forms of Aβ are soluble oligomers and intermediate amyloids (Walsh and Selkoe, 2007), which can cause synaptic loss, neurotoxicity, neuron apoptosis, inflammation and mitochondrion dysfunction (Walsh et al., 2005; Hauptmann et al., 2006; Klyubin et al., 2008; Reddy and Beal, 2008). Tau are microtubule-associated proteins which stabilize the microtubule and promote vesicle transportation. In neurons, the microtubules are essential for the maintenance of neuronal structure, axonal transportation and neuronal plasticity (Lindwall and Cole, 1984). Highly phosphorylated Tau can lose its stabilization ability and may start to self-form NFTs. Tau are phosphorylated by types of kinases, and the phosphate residues are removed by phosphatases (Iqbal et al., 2005). The imbalance between the hyper-phosphorylated and de-phosphorylated forms of Tau could lead to the formation of NFTs (see Figure 2). Tauopathies are considered to be an indicator of the severity of AD.

MicroRNAs, a class of non-coding RNAs, have been acknowledged as important regulators for post-transcriptional gene expression by either repressing translation or degrading target mRNAs (Maoz et al., 2017). Since their discovery, microRNAs have been identified as the regulators most frequently implicated in many critical biological events, such as development, growth, differentiation and neurodegenerative processes (Huang et al., 2011; Hammond, 2015). One microRNA could target numerous genes and one gene could be regulated by multiple microRNAs, making microRNAs a potential tool to investigate multifactorial diseases, for example AD (Iqbal and Grundke-Iqbal, 2010). The study about examining the 13-brain associated microRNAs abundance in human hippocampus samples from fetal and aged adults, age-matched AD patients suggest that misregulation and alterations of specific microRNAs might make contributions to the process of AD (Lukiw, 2007). Afterward, tremendous researches have demonstrated the alterations of several microRNAs between AD patients and age-matched control, further prove that microRNAs might play an important role in the pathogenesis of AD (Goodall et al., 2013). In this review, we will focus on the microRNAs involved in the two most accepted hypotheses of AD pathogenesis: the Aβ hypothesis and the Tau hypothesis.

## MicroRNAs Involved in the Aβ Hypothesis

Since microRNAs have been used to research AD, accumulating microRNAs have been identified as regulators in the process of Aβ production and clearance. The amyloid cascade hypothesis mentions that the imbalance between the production and clearance of Aβ42 could trigger synaptic loss and neurotoxicity. Section “MicroRNAs Involved in the Aβ Hypothesis” describes the microRNAs implicated in the Aβ hypothesis (as shown in Figure 1 and Tables 1, 2).

**Table 1:** A summary of the microRNAs involved in AD.

MicroRNAs	Main model system	Observed main effect	Target mRNA	Reference
MiR-106a	HEK-293 cell.	Overexpressed miR-106a results in translational repression of APP mRNA and significantly reduces APP protein levels.	APP	Patel et al., 2008
MiR-106b	AD brain; HeLa cells; Neuro2A and human SK-N-SH cells; mouse developing brain and primary mouse cortical neurons and glutamatergic neurons derived from mouse embryonic stem cells.	MiR-106b expression decreased in AD brains; and overexpression of miR-106b affected relative luciferase expression cloned with APP and repressed APP protein level. The reduction of miR-106b during brain development in mouse is well correlated with the upregulation of APP protein levels; the correlation between APP and miR-106b also confirmed in these cells.	APP	Hébert et al., 2009
	In the temporal cortex of AD patients and SH-SY5Y cells.	MiR-106b decreased with Fyn increased; and overexpression of miR-106b inhibited Aβ induced tau phosphorylation at Tyr18 and the expression of Fyn. Fyn was a direct target gene of miR-106b.	Fyn	Liu et al., 2016b
MiR-520c	HEK-293 cell.	Overexpressed miR-520c results in translational repression of APP mRNA and significantly reduces APP protein levels.	APP	Patel et al., 2008
MiR-20a and miR-17-5p	HeLa cells; Neuro2A and human SK-N-SH cells; mouse developing brain and primary mouse cortical neurons and glutamatergic neurons derived from mouse embryonic stem cells.	Overexpression of miR-20a and miR-17-5p affected relative luciferase expression cloned with APP and repressed APP protein levels; blocking endogenously miR-20a and miR-17-5p could increase APP protein levels; the reduction of miR-20a and miR-17-5p during brain development in mouse is well correlated with the upregulation of APP protein levels; the correlation between APP and miR-20a and miR-17-5p also confirmed in these cells.	APP	Hébert et al., 2009
MiR-101	Rat hippocampal neurons.	miR-101 is a negative regulator of APP expression.	APP	Vilardo et al., 2010
MiR-16	SAMP8 mice-AD model and BALb/c mice embryos.	APP is the target of miR-16 and low expression of miR-16 could potentially lead to APP protein accumulation in AD mice.	APP	Liu et al., 2010
MiR-153	APPswe/PSΔE9 murine model and miR-153 transgenic mouse model.	MiR-153 were decreased at early and late stage of AD; miR-153 downregulated the expression of APP and APLP2 protein.	APP	Liang et al., 2012
	Cultured human fetal brain cells and human AD brain specimens.	MiR-153 physiologically inhibited expression of APP and miR-153 levels were reduced with elevated APP levels.	APP	Long et al., 2012
MiR-29a/b1	AD brain and HEK293 cells.	Reduction of miR-29a/b1 correlated with high levels of BACE1; miR-29a/b1 negatively regulate BACE1 activity and Aβ formation.	BACE1	Hébert et al., 2008
MiR-29c	Sporadic AD brains; SH-SY5Y cells and HEK293 cells.	MiR-29c expression decreased with upregulated of BACE1 in mRNA and protein levels with elevated APPβ accumulation in sporadic AD brains; miR-29c targeted the 3′UTR of BACE1, reduced the BACE1 expression and downregulated the APPβ accumulation *in vitro*.	BACE1	Lei et al., 2015
MiR-107	AD patient brain tissues and cell culture reporter assays.	MiR-107 levels decreased significantly even in the earliest stages of pathology with the increase of mRNA levels of BACE1. BACE1 is the target of miR-107.	BACE1	Wang et al., 2008
	Intraventricular injection in mice.	MiR-107 mimic reversed the impairments of spatial memory and LTP and the loss of pyramidal neurons caused by Aβ neurotoxicity.		Shu et al., 2018
MiR-298/328	N2a cells and NIH- 3T3 cells.	MiR-298/328 exert regulatory effects on BACE1 protein expression	BACE1	Boissonneault et al., 2009
MiR-195	SAMP8 mice and N2a/APP cells	MiR-195 negatively related with BACE1 protein level; overexpression of miR-195 decreased the level of Aβ.	BACE1	Zhu et al., 2012
MiR-124	PC12 cells and primary hippocampal neurons.	BACE1 could be negatively regulated by miR-124 and the expression of BACE1 was correlated with cell death induced by Aβ neurotoxicity.	BACE1	Fang et al., 2012
	AD patients and Tg2576mice.	MiR-124 increased in AD patients and Tg2576 AD mice model; overexpression of miR-124 or knockdown of PTPN1 recapitulated AD like phenotypes in mice; rebuilding the miR-124/PTPN1 pathway could restore synaptic fail and memory deficits.	PTPN1	Wang X. et al., 2018
MiR135a	APP/PS1 transgenic mice.	MiR-135a downregulated in hippocampi from APP/PS1 transgenic mice and repressed the expression and activity of BACE1.	BACE1	Liu et al., 2014
MiR-200b	APP/PS1 transgenic mice.	MiR-200b downregulated in hippocampi from APP/PS1 transgenic mice and repressed the expression of APP.	APP	Liu et al., 2014
	Blood-derived monocytes (BDMs) and monocyte-derived macrophages (MDMs).	The chemokine/chemokine receptor CCL2/CCR2 axis was impaired in BDMs from AD and miR-200b upregulated in these cells.		Guedes et al., 2016
MiR-339-5p	AD patients brains; human glioblastoma cells and human primary brain cultures.	MiR-339-5p reduced in AD patients brains; miR-339-5p can target BACE1 and inhibited BACE1 protein expression in human glioblastoma and primary brain cultures.	BACE1	Long et al., 2014
MiR-132	MiR-132/212 knockout mice and luciferase reporter system in Neuro2a cells.	Deletion of miR-132/212 could cause abnormal tau metabolism, accentuate tau hyperphosphorylation and tau aggregation. Tau is a direct target of miR-132.	Tau	Smith et al., 2015
	APPPS1 mice.	MiR-132 loss de-represses ITPKB and aggravates amyloid and Tau pathology in AD brain.	ITPKB	Salta et al., 2016
	Triple transgenic AD mice.	Genetic deletion of miR-132/212 promotes Aβ production and amyloid plaque formation; the modulation of miR-132 or Sirt1 can directly regulate Aβ production in cells.	Sirt1	Hernandez-Rapp et al., 2016
	Primary human neurons and neural cells.	MiR-132/212 disturbs the balance of *S*-nitrosylation and induces tau phosphorylation in a NSO1-dependent way.	NSO1	Wang et al., 2017
	Primary neurons	Aβ25–35 exposure decreased miR-132 expression and elevated the expression of PTEN and FOXO3.	PTEN and FOXO3.	Zhao et al., 2018
	Mouse and human wild-type neurons, and P301S Tau transgenic mice.	MiR-132 provides neuroprotection for tauopathies via regulating the tau modifiers acetyltransferase EP300, kinase GSK3β, RNA-binding protein Rbfox1 and proteases Calpain2 and Caspases 3/7.		El Fatimy et al., 2018
MiR212	MiR-132/212 knockout mice and Neuro2a cells.	Deletion of miR-132/212 could cause abnormal tau metabolism, accentuate tau hyperphosphorylation and tau aggregation.		Smith et al., 2015
	Triple transgenic AD mice.	Genetic deletion of miR-132/212 promotes Aβ production and amyloid plaque formation.		Hernandez-Rapp et al., 2016
	Primary human neurons and neural cells.	miR-132/212 disturbs the balance of *S*-nitrosylation and induces tau phosphorylation in a NSO1-dependent.		Wang et al., 2017
MiR-135b	AD patients peripheral blood samples, hippocampal cells and SAMP8 mice.	MiR-135b has a neuroprotective role via directly targeting of BACE1.	BACE1	Zhang et al., 2016
MiR-98-5p	SH-SY5Y, SK-N-SH, and HEK 293 cells.	Downregulation of miR-98-5p alleviated Aβ-induced viability inhibition and decreased the levels of Aβ via modulating SNX6 expression.	SNX6	Li Q. et al., 2016
MiR-186	Neuronal cells.	MiR-186 suppresses BACE1 expression.	BACE1	Kim et al., 2016
	Human HeLa and HEK293-APPSw cells.	MiR-186 regulates Aβ production via targeting Nicastrin.	Nicastrin	Delay et al., 2014
MiR-34a	Murine microglial cell.	MiR-34a drives the down-regulation of the amyloid sensing and clearance receptor protein TREM2.		Zhao et al., 2016
	AD patients temporal cortex and 3xTg-AD mouse model.	Increased miR-34a expression correlates with the repression of its target genes involved in synaptic plasticity, oxidative phosphorylation and glycolysis.		Sarkar et al., 2016
	Short post-mortem interval (PMI) sporadic AD brain.	Upregulated miR-34a and miR-146a down-regulate mRNA targets involved in synaptogenesis (SHANK3), phagocytosis deficits and tau pathology (TREM2), inflammation (CFH) and amyloidogenesis (TSPAN12).		Jaber et al., 2017
	APP/PS1 mice	miR-34a deficiency promotes cognitive function by increasing synaptic plasticity via AMPA and NMDA receptors.		Xu et al., 2018
MiR-146a	SH-SY5Y cells and 5xFAD mice	Dysregulation of miR-146a biogenesis contributes to tau hyperphosphorylation and AD pathogenesis via repressing ROCK1 expression	ROCK1	Wang et al., 2016
	Short post-mortem interval (PMI) sporadic AD brain.	Upregulated miR-34a and miR-146a down-regulate mRNA targets involved in synaptogenesis (SHANK3), phagocytosis deficits and tau pathology (TREM2), inflammation (CFH) and amyloidogenesis (TSPAN12).		Jaber et al., 2017
MiR-34c	Rat primary hippocampal neurons and N2a cells; and lateral ventricle injection in mice.	MiR-34c downregulation ameliorates Aβ-induced synaptic failure and memory deficits by targeting VAMP2.	VAMP2	Hu et al., 2015
MicroRNA-125b	Primary hippocampal and cortical neuron cultures; and mice hippocampus injection.	Overexpression of miR-125b induces tau hyperphosphorylation and cognitive deficits in AD.		Banzhaf-Strathmann et al., 2014
	N2a APPSwe/Δ9 cells model.	MiR-125b may regulate AD and neuronal cell growth and apoptosis via regulating inflammatory factors and oxidative stress by SphK1.	SphK1	Jin et al., 2018
MiR-330	AD mouse model and neuron cells.	MiR-330 exert protective effects on Aβ production, oxidative stress and mitochondrial dysfunction by targeting VAV1 via the MAPK signaling pathway.	VAV1	Zhou et al., 2018
MiR-24/455	Human HeLa and HEK293-APPSw cells.	MiR-24/455 regulate Aβ production via targeting Nicastrin.	Nicastrin	Delay et al., 2014
MiR-128	Monocytes from AD patients	MiR-128 upregulation correlates with impaired Aβ degradation.		Tiribuzi et al., 2014
	BDMs and MDMs.	The chemokine/chemokine receptor CCL2/CCR2 axis was impaired in BDMs from AD and miR-128 upregulated in these cells.		Guedes et al., 2016
MiR-155, miR-154 and miR-27b	BDMs and MDMs.	The chemokine/chemokine receptor CCL2/CCR2 axis was impaired in BDMs from AD and miR-155, miR-154, and miR-27b upregulated in these cells.		Guedes et al., 2016
MiR-1908	Peripheral blood cells from AD patients and cultured cell lines.	miR-1908 up-regulation impairs amyloid clearance by targeting ApoE	ApoE	Wang Z. et al., 2018
MiR-302	AD blood cells and SK-N-MC cells.	MiR-302 attenuates Aβ induced neurotoxicity through activation of Akt signaling.	PTEN	Li Q. et al., 2016
MiR-137	Primary mouse cortical neurons and N2a cells.	MiR-137 attenuates Aβ-induced neurotoxicity through inactivation of NF-kB pathway.	TNFAIP1	He et al., 2017
MiR-188-5p	5xFAD mouse model and primary hippocampal neuron cultures.	Replenishment of miR-188-5p restores the synaptic and cognitive deficits.		Lee et al., 2016
MiR-10a	Hippocampal neurons and AD rat model.	MiR-10a restrains synapse remodeling and neuronal cell proliferation while promoting apoptosis in AD rats via inhibiting BDNF-TrkB signaling pathway.	BDNF	Wu et al., 2018
MiR-26b	Rat primary post-mitotic neurons.	Upregulated miR-26b activates cycle entry, tau phosphorylation and apoptosis in postmitotic neurons.	Rb1	Absalon et al., 2013
MiR-922	SH-SY5Y and HEK-293T cells	MiR-922 promotes tau phosphorylation by downregulating UCHL1 expression.	UCHL1	Zhao et al., 2014
MiR-138	N2a/APP and HEK293/tau cells.	MiR-138 promotes tau phosphorylation by targeting RARA.	RARA	Wang et al., 2015
MiR-124-3p	N2a/APP695swe cells.	MiR-124-3p attenuates hyperphosphorylation of tau protein induced apoptosis via caveolin-1-PI3K/Akt/GSK3β pathway.	Caveolin-1	Kang et al., 2017

**Table 2:** microRNAs modulating Aβ plaques formation and tau phosphorylation.

mRNA targets	Involved miRNAs	Main model system	Main effect	Reference
APP	miR-106a and miR-520c	HEK-293 cell	Overexpressed miR-106a/520c results in translational repression of APP mRNA and significantly reduces APP protein levels.	Patel et al., 2008
	miR-106b, miR-20a, miR-17-5p	AD brain and HeLa cells; Neuro2A and human SK-N-SH cells; mouse developing brain; primary mouse cortical neurons and glutamatergic neurons;	miR-106b, miR-20a, miR-17-5p expression decreased in AD brains; overexpression of miR-106b, miR-20a, miR-17-5p affected relative luciferase expression cloned with APP. Overexpression of miR-106b, miR-20a, miR-17-5p could repress APP protein level. The reduction of miR-20a, miR-17-5p and miR-106b during brain development in mouse is well correlated with the upregulation of APP protein levels; the correlation between APP and miR-20a, miR-17-5p, and miR-106b also confirmed in these cells.	Hébert et al., 2009
	miR-101	In rat hippocampal neurons	miR-101 is a negative regulator of APP expression.	Vilardo et al., 2010
	miR-16	In SAMP8 mice-AD model and BALb/c mice embryos.	APP is the target of miR-16 and low expression of miR-16 could potentially lead to APP protein accumulation in AD mice.	Liu et al., 2010
	miR-153	In the cultured human fetal brain cells and human AD brain specimens.	miR-153 physiologically inhibited expression of APP; miR-153 levels were reduced with elevated APP levels.	Long et al., 2012
		In APPswe/PSΔE9 murine model and miR -153 transgenic mouse model.	miR-153 were decreased at early and late stage of AD and downregulated the expression of APP and APLP2 protein.	Liang et al., 2012
	miR-200b	APP/PS1 transgenic mice.	miR-200b downregulated in hippocampi from APP/PS1 transgenic mice and repressed the expression and activity of APP.	Liu et al., 2014
BACE1	miR-29a/b1	In AD brain and HEK293 cells.	Reduction of miR-29a/b1 correlated with high levels of BACE1 and miR-29a/b1 negatively regulate BACE1 activity and Aβ formation.	Hébert et al., 2008
	miR-29c	In sporadic AD brains; SH-SY5Y cells and HEK293 cells.	miR-29c decreased with upregulated of BACE1 in mRNA and protein levels, and elevated APPβ accumulation; miR-29c targeted the 3′UTR of BACE1, reduced the BACE1 expression and downregulated the APPβ accumulation *in vitro*.	Lei et al., 2015
	miR-107	AD patients brains tissues and in mice.	miR-107 levels decreased significantly even in the earliest stages of pathology with the increase of mRNA levels of BACE1. BACE1 is the target of miR-107.	Wang et al., 2008
	miR-298/328	In N2a cells and NIH 3T3 cells.	miR-298/328 exert regulatory effects on BACE1 protein expression.	Boissonneault et al., 2009
	miR-195	In SAMP8 mice and N2a/APP cells.	MiR-195 negatively related with BACE1 protein level and overexpression of miR-195 decreased the level of Aβ.	Zhu et al., 2012
	miR-124	In PC12 cells and primary hippocampal neurons.	BACE1 could be negatively regulated by miR-124 and the expression of BACE1 was correlated with cell death induced by Aβ neurotoxicity.	Fang et al., 2012
	miR135a	APP/PS1 transgenic mice.	MiR-135a downregulated in hippocampi from APP/PS1 transgenic mice and repressed the expression and activity of BACE1.	Liu et al., 2014
	miR-339-5p	AD patients brains; human glioblastoma cells and human primary brain cultures.	MiR-339-p reduced in AD patients brains; miR-339-5p can target BACE1 and inhibited BACE1 protein expression in human glioblastoma and primary brain cultures.	Long et al., 2014
	miR-135b	AD patients peripheral blood, SAMP8 mice and hippocampal cells.	MiR-135 has a neuroprotective role via direct targeting of BACE1.	Zhang et al., 2016
	miR-186	In neuronal cells.	miR-186 suppresses BACE1 expression.	Kim et al., 2016
Tau	miR-132	miR-132/212 knockout mice and Neuro2a cells.	Deletion of miR-132/212 could cause abnormal tau metabolism, accentuate tau hyperphosphorylation and tau aggregation; Tau is a direct target of miR-132.	Smith et al., 2015
Fyn	miR-106b	In the temporal cortex of AD patients and SH-SY5Y cells.	miR-106b decreased with Fyn increased; overexpression of miR-106b inhibited Aβ induced tau phosphorylation at Tyr18 and the expression of Fyn. Fyn was a direct target gene of miR-106b.	Liu et al., 2016b

### MicroRNAs Involved in Aβ Production

Mounting evidence has shown that specific microRNAs play a key role in regulating the expression of APP and BACE1, which restrict the production of Aβ.

#### MicroRNAs Regulate APP Expression

Accumulating evidence demonstrates that increased APP expression could promote Aβ production, resulting in neurotoxicity, synaptic failure and, eventually, dementia (Patel et al., 2008). Many microRNAs have participated in the process of regulating APP expression; for example, Patel et al. (2008) found that miR-106a/520c could bind the 3′UTR of APP and repress the expression of APP in human cell lines. Later, the Hebert group found that the miR-20 families (miR-20a, miR-17-5p, and miR-106b) could also regulate APP expression via the binding of APP 3′UTR. In addition, in accordance with miR-20 overexpression in neuron cells, the expression level of APP decreased (Hébert et al., 2009). Similarly, miR-101 (Vilardo et al., 2010), miR-16 (Liu et al., 2010), and miR-153 (Long et al., 2012) have also been identified as negative regulators of APP expression both *in vitro* and *in vivo*. Moreover, the low level of miR-153 contributed to the accumulation of Aβ in sporadic AD patients.

#### MicroRNAs Regulate BACE1 Expression

The division of APP by BACE1 is the first and rate-limit step for Aβ formation, and upregulated BACE1 expression levels and enzymatic activities have been detected in sporadic AD brains (Yang et al., 2003). Several microRNAs regulating BACE1 expression and activity have been found; for example, the miR-29 family implicated in regulating BACE1 expression has been well studied. Hébert et al. (2008) performed the microRNAs expression profiles of sporadic AD patients, and found that the expression levels of the miR29a/b1 cluster decreased significantly with high levels of BACE1 expression in sporadic AD brains. Further analysis showed that miR-29a/b-1 could regulate BACE1 expression *in vitro* and *in vivo*. Moreover, it was validated that decreased levels of miR-29a/b-1 could promote the production of Aβ and contribute to the pathogenesis of AD (Hébert et al., 2008). Afterwards, miR-29c, another miR-29 family member, was also found to be downregulated with abnormally high levels of BACE1 in sporadic AD brains; additional validation experiments found that overexpressed miR-29c could induce BACE1 downregulation in human neuroblastoma SH-SY5Y cells via the binding of BACE1 3′UTR (Lei et al., 2015).

Another well studied microRNA is miR-107. The expression of miR-107 decreased significantly with BACE1 increased in AD patients. Then, it was revealed that miR-107 regulated the expression of BACE1 through recognizing and binding the 3′UTR of BACE1 in a cell culture reporter assay (Wang et al., 2008). Afterwards, Nelson and Wang also demonstrated that miR-107 levels negatively correlated with BACE1 mRNA levels, leading to Aβ accumulation (Nelson and Wang, 2010). Moreover, some studies have shown that miR-107 was implicated in preventing the neurotoxicity and blood–brain-barrier dysfunction induced by Aβ (Liu et al., 2016a; Shu et al., 2018), making miR-107 a potential drug target for researchers (Parsi et al., 2015).

Other negative regulators of BACE1 also include miR-298/328 (Boissonneault et al., 2009), microRNA-195 (Zhu et al., 2012), miR-135a (Liu et al., 2014), microRNA-135b (Zhang et al., 2016), miR-339-5p (Long et al., 2014), and miR-186 (Kim et al., 2016), all of which negatively correlated with BACE1 expression and exerted regulatory effects by binding the 3’UTR of BACE1.

#### Other MicroRNAs Involved in Aβ Production

Recent cases have reported that the miR-132/212 cluster plays an important role in Aβ production; for example, Hernandez-Rapp et al. (2016) found that levels of microRNA-132/212 cluster decreased in AD patients, and the deficiency of microRNA-132/212 could promote Aβ production and senile plaque deposition in AD triple transgenic mice. In another study, Salta et al. (2016) also confirmed that the loss of miR-132 could promote β amyloid accumulation through regulating inositol 1,4,5-trisphosphate 3-kinase B-ITPKB in AD mouse models. Li Q. et al. (2016) found that miR-98-5p could repress the expression of sorting nexin6 (SNX6), which is involved in increasing the levels of Aβ40, Aβ42, BACE1, soluble amyloid precursor protein β (sAPPβ), and membrane-associated APP β-carboxyl terminal fragments in 293T and SK-N-SH cells. Other microRNAs implicated in Aβ production include miR-34a, miR-146 (Jaber et al., 2017), microRNA-125b (Jin et al., 2018), miR-330 (Zhou et al., 2018), miR-24, miR-186, and miR-455 (Delay et al., 2014). All these provide further evidence that specific microRNAs play a critical role in the pathogenesis of AD.

### MicroRNAs Involved in Aβ Clearance

Aβ deposits result from an imbalance between the production and clearance of β amyloid peptides. Thus, the dysfunctional clearance of Aβ could also contribute to the accumulation of Aβ peptides. As such, in Section “MicroRNAs Involved in Aβ Clearance” we describe a number of microRNAs involved in Aβ clearance.

The endosomal–lysosomal system degrades accumulated proteins and functions as a protective factor in the central nervous system. Tiribuzi et al. (2014) found that upregulated miR-128 could impair the clearance of Aβ through the targeting of lysosomal system enzymes in monocytes in sporadic AD patients. Aβ degradation improved when miR-128 was inhibited in monocytes from AD patients (Tiribuzi et al., 2014). Another microRNA involved in Aβ clearance was miR-34a which, when upregulated in AD patients, repressed the clearance of Aβ by inhibiting the expression of TREM2 (Zhao et al., 2016). TREM2, a myeloid/microglial cell surface amyloid sensor-receptor implicated in recognizing and digesting Aβ and extracellular amyloidogenic debris, plays a key role in the clearance of over-expressed Aβ (Wang et al., 2015; Song et al., 2016; Ulrich and Holtzman, 2016). The expression of TREM2 also correlated with blood-derived monocytes (BDMs) (Guedes et al., 2016), which have been implicated in the clearance of β amyloid deposits in AD brains (Michaud et al., 2013). Immune-related microRNAs (miR-155,-154,-200b,-27b, and 128) which, when expressed differentially in CCL2/CCR2 (chemokine/chemokine receptor) axis impaired BDMs, reportedly participate in the process of Aβ clearance mediated by BDMs (Guedes et al., 2016). Additionally, several researchers have revealed that differentially expressed apoE isoforms also play a role in Aβ clearance (Deane et al., 2008; Liu et al., 2013). It has been validated that miR-1908 could inhibit ApoE expression in mRNA and protein levels in human macrophage cell line THP-1 and astrocytoma cell line U87. In AD patients, the level of miR-1908 was negatively related to ApoE expression, which suggested that miR-1908 played a crucial role in inhibiting Aβ clearance through repressing ApoE expression (Wang Z. et al., 2018).

### MicroRNAs Involved in Aβ Induced Neurotoxicity

Aβ could induce neurotoxicity, and this section describes several microRNAs involved in this process. In 2016, it was discovered that miR-302 could restore Aβ induced neurotoxicity via the PTEN/Akt/Nrf2/HO-1 pathway in AD neurons (Li H. H. et al., 2016). He et al. (2017) found that miR-137 could reduce Aβ induced neurotoxicity by inhibiting the process of NF-κB via repressing TNFAIP1 expression in N2a cells. It has been reported by many groups that miR-132 decreased significantly in AD brains. Zhao et al. (2018) recently discovered that the overexpression of miR-132 in cultured cortical neurons could inhibit the neurotoxicity induced by Aβ via the miR-132/PTEN/AKT/FOXO3a pathway. In the same year, Shu et al. (2018) found that miR-107 could block Aβ induced neurotoxicity in mice; they injected miR-107 mimics, which could intraventricularly generate native miR-107, into mice and found that miR-107 could restore spatial memory impairment and pyramidal neurons loss caused by Aβ. It was reported that MiR-124 could alleviate neurotoxicity via regulating the expression of BACE-1 in AD cellular models (Fang et al., 2012). These microRNAs provide us with a new way to understand the pathogenesis mechanisms of AD.

### MicroRNAs Involved in the Aβ Induced Synaptic Failure

Aβ could lead to the synaptic failure and numerous microRNAs involved in this process. It was reported that the level of miR-34c increased significantly in the transgenic mice model, AD patients and Aβ exposed neuron cells. Further investigation demonstrated that inhibiting the expression of miR-34c could rescue the synaptic dysfunction and memory loss induced by Aβ by up-regulating the expression of VAMP2 (Hu et al., 2015). Additionally, Sarkar et al. (2016) identified that miR-34a, another miR-34 family member, could target multiple genes involved in neuronal synaptic deficiency and energy metabolism. Moreover, further research found that the loss of miR-34a could improve cognitive functions through rescuing synaptic dysfunction by repressing the expression of AMPA and NMDA receptors (Xu et al., 2018).

Lee et al. (2016) found that miR-188-5p could restore the synaptic dysfunction and cognitive impairment caused by Aβ in transgenic mice; they also observed that the overexpression of miR-188-5p could alleviate the decrease of dendritic spine density in primary hippocampal neurons exposed to Aβ. Additionally, Wu et al. (2018) found that miR-10a was also a negative regulator in synapse remodeling and cell proliferation as a result of a reduction in BDNF-TrkB signals in AD rats. They confirmed that BDNF was a target of miR-10a using a dual luciferase gene reporter assay (Wu et al., 2018).

Recently, a novel pathway, the miR-124/PTPN1 pathway, has been reported to involve in synaptic transmission deficits. The level of miR-124 increased significantly with the expression of its target, PETN1, decreased dramatically in AD brains. The overexpression of miR-124 or the knockdown of PTPN1 in mice could induce AD-like phenotypes, containing synaptic transmissions and plasticity deficits. Moreover, the inhibition of miR-124 expression or the over-expression of PTPN1 could alleviate the synaptic deficits in AD model mice (Wang X. et al., 2018).

A noticeable thing is that Aβ could induce the synaptic loss, changes in neurotrophin and inflammations, conversely, these deficits could affect the clearance and degradation of Aβ. NF-κB, a pro-inflammatory transcription factor, could be activated by Aβ, and the activation of NF-κB could lead to the upregulated of six inducible miRNAs: miR-7, miR-9, miR-34a, miR-125b, miR-146a, and miR-155 (Zhao et al., 2015). Those NF-κB sensitive upregulated miRNAs in AD brain contributed much to the neuropathological characteristics. For example, upregulated miR-125b may impair the Aβ clearance via repressing the TREM2 expression, induce chronic innate-immune and inflammatory signaling by inhibiting the expression of CFH (complement factor H), repress neurotransmitter package and release, and decrease the neurotrophin within the brain through regulating the expression of SYN-2, 15-LOX, and VDR (synapsin-2, 15-lipoxygenase and vitamin D receptor). Similarly, miR-146a may be responsible for promoting amyloidogenesis through regulating the expression TSPAN12 (tetraspanin12) and/or failing to modulate the NF-κB through regulating IRAK-1 and IRAK-2 (interleukin-1 receptor associated kinase) (Zhao et al., 2015).

## MicroRNAs Involved in the Tau Phosphorylated Imbalance

Neurofibrillary tangles were largely composed of hyper-phosphorylated Tau as a result of the imbalance between Tau phosphorylation and de-phosphorylation through regulating the expressions and activities of a number of related kinases and phosphatases (Ballatore et al., 2007). In Section “MicroRNAs Involved in the Tau Phosphorylated Imbalance,” we describe several microRNAs involved in the process of phosphorylation and de-phosphorylation (as shown in Figure 2 and Tables 1, 2).

It is reported that the expression levels of miR-125b are increased significantly in AD patients (Banzhaf-Strathmann et al., 2014; Ma et al., 2017; Jin et al., 2018). In both neuron cells and in mice, over-expression of miR-125b induced Tau hyper-phosphorylation through the targeting of phosphatases DUSP6 and PPP1CA, while inhibition of miR-125b induced a reduction in Tau phosphorylation and kinase expression/activity (Banzhaf-Strathmann et al., 2014). Moreover, Ma et al. (2017) found that overexpression of miR-125b could induce cell apoptosis and Tau hyper-phosphorylation by activating the cyclin-dependent kinase 5 (CDK5) and p35/25 in neuron cells. MiR-125b might be implicated in this process through targeting the forkhead box Q1(FOXQ1) directly (Ma et al., 2017).

Smith et al. (2015) found that the expression of Tau could be directly regulated by miR-132, and the deletion of miR-132/212 in mice could increase the expression, phosphorylation and accumulation of Tau. They also revealed that the delivery of miR-132 mimics into AD mice could reduce the metabolism of Tau (Smith et al., 2015). Wang et al. (2017) also reported that the downregulation of miR-132/212 could promote Tau phosphorylation and break the imbalance between Tau phosphorylation and de-phosphorylation via the NOS1-dependent pathway in primary human neurons and neural cells. miR-132 has been recognized in numerous studies as one of the most significant downregulated microRNAs, and it has been implicated in the process of Tau hyper-phosphorylation through the targeting of EP300, GSK3b, Rbfox1, proteases Calpain2 and caspases 3/7 (El Fatimy et al., 2018).

The expression level of miR-138 is up-regulated in AD patients. Wang et al. (2015) found that miR-138 could directly target the retinoic acid receptor alpha which could repress glycogen synthase kinase-3b (GSK-3β) activity. They further confirmed that miR-138 could promote Tau phosphorylation via the RARA/GSK-3β pathway in N2a/APP and HEK293/tau cells (Wang et al., 2015). The level of miR-26b also increased significantly in the pathological areas of postmortem AD brains. It was confirmed that the elevated levels of miR-26b contributed to the pathology of AD through cell cycle entry, Tau hyper-phosphorylation and apoptosis in post-mitotic neurons (Absalon et al., 2013).

MicroRNA-922 was also reported to contribute to the pathogenesis of AD via regulating the expressions of UCHL1. The levels of UCHL1 were decreased in sporadic AD patients, and the overexpression of UCHL1 rescued the synaptic and cognitive function in AD model mice. And in the AD model mice, the expression levels of miR-922 were upregulated with UCHL1 protein levels decreased. Zhao et al. (2014) found that UCHL1 was one target of miR-922, and the phosphorylation levels of Tau were negatively correlated with the expressions of UCHL1. Thus, they suggested that miR-922 involved in the pathogenesis of AD through regulating the phosphorylation levels of Tau by targeting UCHL1 (Zhao et al., 2014).

The expression levels of microRNA-146a was increased in AD patients’ brains, and the dysregulation of microRNA-146a biogenesis was involved in the tau hyperphosphorylation and AD pathogenesis. In neural cells, it was confirmed that miR-146a targeted the coiled-coil containing protein kinase1 (ROCK1) directly, and the inhibition of ROCK1 could induce abnormal tau phosphorylation, which was related with the low phosphorylation levels of the phosphatase and tensin homolog (PTEN). Additionally, the ROCK1 colocalised with the hyperphosphorylated tau in the early neurofibrillary tangles with decreased ROCK1 protein levels in AD patients. Moreover, in 5xFAD mice, the inhibition of miR-146a through intra-hippocampal delivery induced upregulated ROCK1 protein levels and repressed tau hyperphosphorylation, and restored memory function partly (Wang et al., 2016).

Similarly, the expression level of miR-106b was downregulated with the upregulation of Fyn in the temporal cortex of AD patients. In tau stably expressed SH-SY5Y cells (SH-SY5Y/tau), the overexpression of miR-106b inhibited Aβ42 induced tau phosphorylation at Tyr18 without changes at Ser396/404. It was validated that miR-106b could target Fyn directly and repress the protein levels of Fyn in SH-SY5Y cells. The phosphorylation level of tau at Tyr18 was decreased when Fyn was knockdown, and the inhibitory effects could be rescued when the expression of miR-106b was inhibited. Thus, miR-106b might inhibit Aβ42 induced tau phosphorylation at Tyr18 via regulating the expression of Fyn (Liu et al., 2016b).

The expression levels of miR-124b was decreased in the brain of AD patients and in N2a/APP695swe cells. The overexpression of miR-124-3p could rescue cell apoptosis and attenuate the abnormal hyperphosphorylation of tau with the upregulation of Caveolin-1, phosphoinositide 3-kinase (PI3K), phosphor-Akt (Akt-Ser473)/Akt, phosphor-glycogen synthase kinase-3 beta (GSK-3β-Ser9)/GSK-3β in N2a/APP695swe cells. It was suggested that miR-124-3p could inhibit abnormal hyperphosphorylation of tau through targeting Caveolin-1 and regulating the pathway Caveolin-1-PI3K/Akt/GSK-3β (Kang et al., 2017).

### Perspectives

Notably, the pathological functions of multiple miRNAs regulated genes in AD-affected brain are overlapped and highly interactive, and the effects of interactive and overlapped functions network are greater than the individual one. Another noticeable problem is that specific brain enriched miRNAs are relatively labile and short-lived (Sethi and Lukiw, 2009), they may be subject to degradation especially in the highly oxidative environment of an AD brain. Thus, down-regulated microRNAs may be an artifact of the degenerative aspects of the disease. While the up-regulated miRNAs and their down-regulated mRNAs targets are a better bet to study especially in post-mortem tissues (Guo et al., 2010).

In 2018, the National Institute of Aging and Alzheimer’s Association proposed that “Alzheimer’s Disease” was a process of accumulated neuropathological changes, then defined as “AD” by biomarkers *in vivo* or by postmortem examination, not clinical symptoms (Jack et al., 2018); thus, the search for reliable, effective and timely biomarkers would be of significant value. The most commonly used biomarkers include CSF (cerebrospinal fluid) Aβ42 or Aβ42/Aβ40 ratio, amyloid PET, CSF phosphorylated Tau, CSF total Tau, Tau PET, anatomic MRI and FDG PET, which all focus on Aβ and Tau in bio fluids and neuroimaging techniques (Femminella et al., 2015; Jack et al., 2018). However, none of them could individually diagnose AD, and these methods still arrived too late for early and effective intervention. In recent decades, many studies have shown that microRNAs are implicated in the pathogenesis of AD (described in sections “MicroRNAs Involved in the Aβ Hypothesis” and “MicroRNAs Involved in the Tau Phosphorylated Imbalance” and summarized in Tables 1, 2), and alterations of microRNAs were found in serum, plasma and CSF, meaning that microRNAs are exceptional candidates for AD biomarkers. Most microRNA biomarker lists were produced by conducting microRNA profiles through qPCR (Dong et al., 2015), some of which are even next generation sequences (Guo et al., 2017), and comparing differentially expressed microRNAs between AD patients and control groups. Although mounting microRNAs have been identified as potential biomarkers for AD diagnosis, few have been validated in more than two studies, making their widespread use difficult (Femminella et al., 2015). It was proved that the expression levels of miR-15, miR-181c, miR-125b in AD CSF were up/down-regulated compared with normal controls in more than two studies, and so these specific microRNAs were suggested to be potential biomarkers for AD (Richardson et al., 2008; Danborg et al., 2014; Tan et al., 2014). Despite this, extensive validation and follow-ups in larger cohorts of patients are still necessary.

There are still no drugs that are both safe and effective for AD use, even though multiple agents have been researched and tested. Studies about microRNAs in AD have provided prominent insights into our understanding of molecular mechanisms, shedding light on potential drugs by targeting specific microRNAs. The regulatory characteristics of microRNAs are sequence-specific and multiple genes regulation, providing an exciting avenue for regulating this complicated disease networks and pathways. Approximately 500 patents were issued and published in United States and European in 2016, suggesting that the therapeutic potentials of microRNAs have attracted a great deal of attention. A number of preclinical studies also have been performed, for example, Parsi et al. (2015) performed a preclinical evaluation and suggested that miR-16 was a good drug candidate for AD. They identified that miR-16 could inhibit the expression of APP and BACE1, repress the production of Aβ and the phosphorylation of Tau in cells. Additionally, the delivery of miR-16 into mice induced a decrease in APP, BACE1, and Tau levels in a region dependent method. Moreover, miR-16 has also been found to associate with oxidative stress and inflammation in AD (Parsi et al., 2015). This successful preclinical study provided support for future microRNA therapy researches. It is important to note that the off target effect of microRNAs should not be ignored at the same time. Although studies about microRNA therapies in AD are still in their infancy, with increasing attention being paid to this field, we believe that researchers will fully understand this at some point, even if there is still a long way to go.

## Author Contributions

MW wrote the manuscript. LQ edited it and BT edited the final version of the manuscript.

## Conflict of Interest Statement

The authors declare that the research was conducted in the absence of any commercial or financial relationships that could be construed as a potential conflict of interest.
